# An agent-based model for collaborative learning to combat antimicrobial resistance: proof of concept based on broiler production in Senegal

**DOI:** 10.1016/j.soh.2023.100051

**Published:** 2023-11-04

**Authors:** Paul Python Ndekou, Archie Drake, Jake Lomax, Michel Dione, Ardiouma Faye, Mohamed Daly Njiemessa Nsangou, Lilian Korir, Elizabeth Sklar

**Affiliations:** a Cheikh Anta Diop University, Dakar 10700, Senegal; b University of Lincoln, Brayford Pool, Lincoln, LN6 7TS, United Kingdom; c Mutate Systems Development, 28a Waterloo Road, Falmouth, England, TR11 3NU, United Kingdom; d International Livestock Research Institute, Rue 18 Cité Mamelles, BP 24265 Ouakam, Dakar, Senegal; e Independent Veterinary Researcher, Rue 8∗23 Medina, BP 5077 Fann, Dakar, Senegal

**Keywords:** Antibiotics, Agent-based model, Poultry, Livestock systems, Policy

## Abstract

Antimicrobial resistance (AMR) is a substantial global One Health problem. This paper reports on initial, proof-of-concept development of an agent-based model (ABM) as part of wider modelling efforts to support collaborations between groups interested in policy development for animal health and food systems. The model simulates AMR in poultry production in Senegal. It simultaneously addresses current policy issues, builds on existing modelling in the domain and describes AMR in the broiler chicken production cycle as seen by producers and veterinarians. This enables implementation and assessment of producer antimicrobial use and infection prevention and control strategies in terms of immediate economic incentives, potentially helping to advance conversations by addressing national policy priorities. Our model is presented as a flexible tool with promise for extension as part of AMR policy development in Senegal and West Africa, using participatory approaches. This work indicates that ABM can potentially play a useful role in fostering counter-AMR initiatives driven by food system actor behaviour in lower- and middle-income countries more generally.

## Highlights

• Antimicrobial resistance (AMR) contributes to production loss and food insecurity.
• Poultry production is a policy priority in West Africa as well as for global AMR.
• An Agent-Based Model (ABM) simulates complex AMR and disease dynamics in Senegal.
• The ABM offers a flexible, extensible environment for relating evidence to policy.
• Participatory ABM helps cut past levels of abstraction for local policy engagement.

## 1 Introduction

AMR is a major threat to global human health, with the latest evidence showing that the highest mortality burden of AMR is in West Africa [1]. Action on AMR is accordingly a growing priority for global health systems, under a One Health approach designed to address food safety as well as zoonoses, with an emphasis on the development of knowledge and evidence through surveillance and research [2,3].

Antimicrobial use (AMU) in poultry production is vitally relevant to the counter-AMR agenda in this context because of this food system’s importance to poverty alleviation and economic development efforts, including from a food security perspective [4]. Unfortunately, too little is known about apparent causal pathways to AMR human health burdens. But there is another more immediate and potentially compelling aspect to the AMR threat from within animal health: the risk that treatment failures will drive production losses and food insecurity [5]. This is the main motivation for efforts to develop relevant socio-technical interventions, for example through research on African farmers' knowledge, attitudes and practices (KAP) [6,7]. KAP studies point both to the need and to the means to combine “top-down” regulatory interventions with “bottom-up” stakeholder-led local adaptation (plus infrastructural change to provide an “enabling environment” in resource-limited contexts), promoting investment in preventative practices relative to antimicrobial treatment to reduce reliance on antimicrobials.

Senegal is committed to action on AMR as a policy priority under the leadership of the High Council for Global Health Security (HCNSSM). The country stands out for its dynamic efforts to understand AMR in animal health. Antimicrobial use and consumption (AMU/C) aspects of livestock production have been a focus of attention for the Ministry of Livestock and other stakeholders, including the Emergency Centre for Transboundary Animal Diseases (ECTAD) supported by the United Kingdom’s Fleming Fund [8]. There is a vigorous animal health AMR research community in Senegal, with a variety of studies considering the poultry sector in particular – for example [[9], [10], [11], [12]]. Various reports have highlighted the increasing use of antimicrobials in the sector, with the resistance of bacteria such as *Echerichia coli* and *Salmonella* to commonly used antibiotics [13]. Recent indications of high threat of drug resistance in Senegal relative to other African countries in the human health context [14], underline “calls for urgent policy intervention” [15].

Still various factors complicate the case for relevant action on AMR, notably obscure effects, including AMU economic incentives in terms of productivity and competitiveness, and the need for market-based solutions, especially given limits to regulatory supervision in animal health. Although economic studies point in this direction, for example for broiler producers in France [16], both animal health and LMIC are under-represented in economic evaluation of AMR-related interventions [17]. Still, new initiatives like the Food & Agriculture Organization of the United Nations (FAO)’s Reduce the Need for Antimicrobials in agrifood systems initiative (RENOFARM) recognize economic objectives as the key to reducing AMU by aiming to improve countries’ agrifood systems transformation through the provision of comprehensive support in the implementation of good production practices [18]. In Senegal, the profile of the poultry sector demonstrates the urgency of the issues and policy development potential. Peri-urban areas of major cities Dakar and Thies exhibit intense livestock-rearing activity, especially chickens, with high animal density in close cohabitation with human settings. The great majority of poultry production is semi-intensive with average rotations of between 300 and 1000 subjects. Public policy plays a vital and active role, underpinning growth with protective measures since 2005 and regulating prices [19,20].

The implication of complex adaptive systems including both human behavioural interventions and disease dynamics make this a potentially fertile subject for *ex ante* ABM [21,22], as an input to ongoing AMR policy discussions in Senegal and beyond. ABM contrasts with more traditional modelling approaches, for example using sets of interrelated differential equations, in that it offers a natural way to describe systems characterized by many levels of interactions, and better capture emergent phenomena [23]. The autonomy of individual agents in ABM allows them to be used as structures for simulation of systems with multiple “loci of control”. Strikingly, ABM has been used extensively to simulate systems in several domains recognized to be constitutive of AMR as a policy problem, including infectious disease itself at both microbiological and population levels, animal herding/flocking (relevant to livestock), economic systems and behavioural policy. AMR ABM efforts can therefore draw together existing conceptual structures.

On the other hand, there are also considerable challenges for model development (including ABM) in the context of AMR in LMIC, especially the lack of relevant data on AMU/C in agriculture and food systems in Africa [24]. Modelling under these circumstances has been conceptualised more as a means of empowering actors lacking social and political information than as a way of making sense of existing data flows [25].

This paper reports on model development to support collaboration between researchers, policymakers, veterinary professionals and producer businesses in efforts to combat antimicrobial resistance (AMR). Its immediate objective is to contribute to current policy dialogue over AMR in poultry production in Senegal and West Africa. Beyond that this research aims to advance agent-based modelling (ABM) as tool in the field, especially in lower- and middle-income countries (LMIC).

## 2 Material and methods

**Fig. 1:**
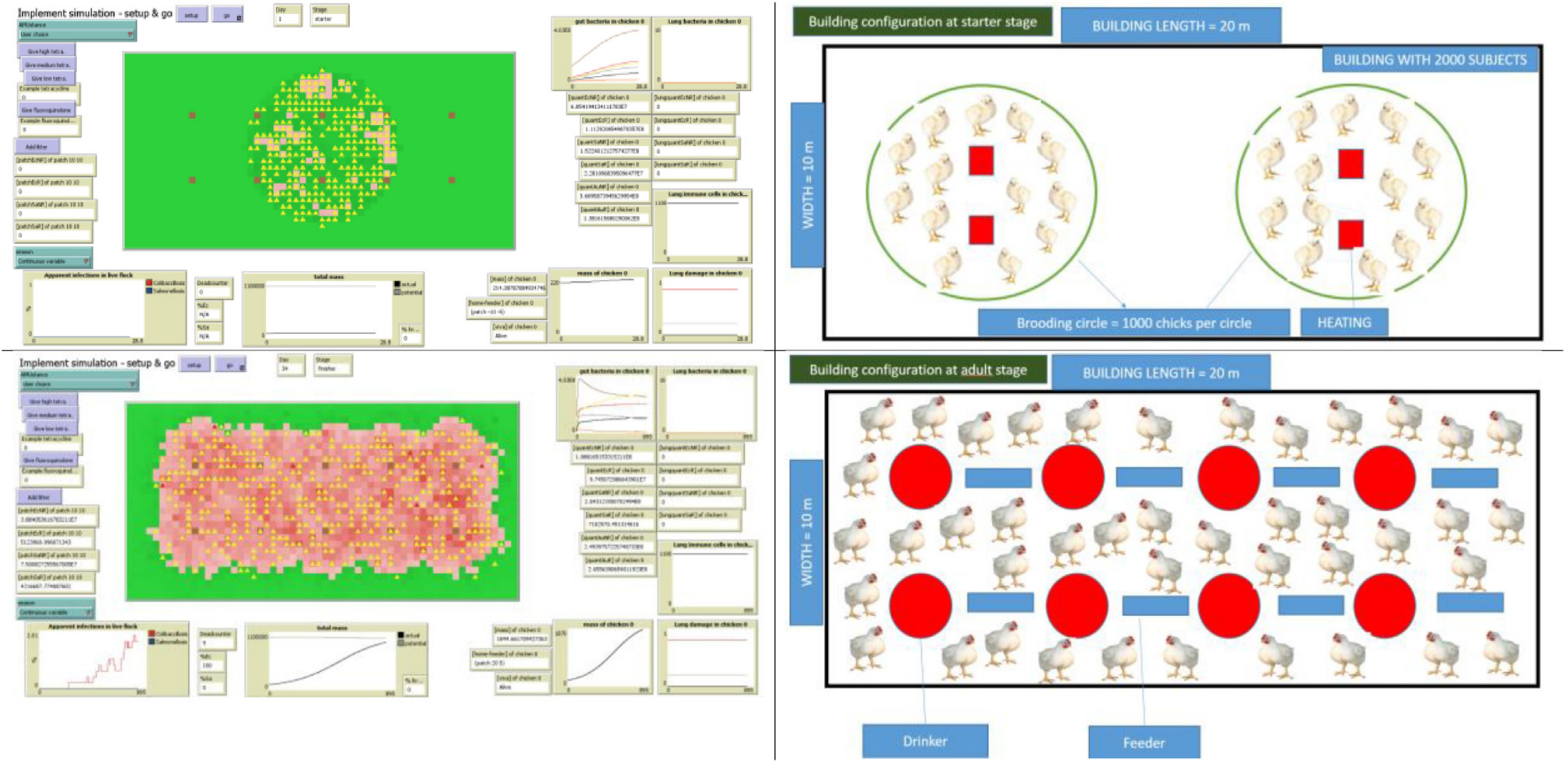
**Model observer interface (left) alongside representation of a standard semi-intensive broiler rearing facility (right).***(Chicks are initially reared in circular starter enclosures (top), moving to occupy the entire rearing facility for later production phases (bottom) (Source: model observer interface/Dr. Njiemessa Nsangou))*.

An ABM was developed in the NetLogo environment, simulating a broiler chicken production environment cycle typical of semi-intensive production characteristic of the Dakar and Thies regions of Senegal. Model code and a description conforming to the Overview, Design concepts, Details (ODD) standard that is a key component of the “TRACE” approach to model development for ABM [26,27] have been made available at (https://github.com/archiedrake/senegalabm.git). The simulation was configured to describe rearing of 500 birds in a standard facility over approximately 42 days, with multiple resistant and non-resistant dynamic bacterial strain populations (including *Escherichia coli* and non-typhoid *Salmonella* spp.) represented in addition to varying concentrations of multiple antimicrobial agents (designed to represent tetracycline and fluoroquinolones). Fig. 1 shows a comparison of the model observer interface with a practitioner’s representation of a standard rearing facility.

**Fig. 2:**
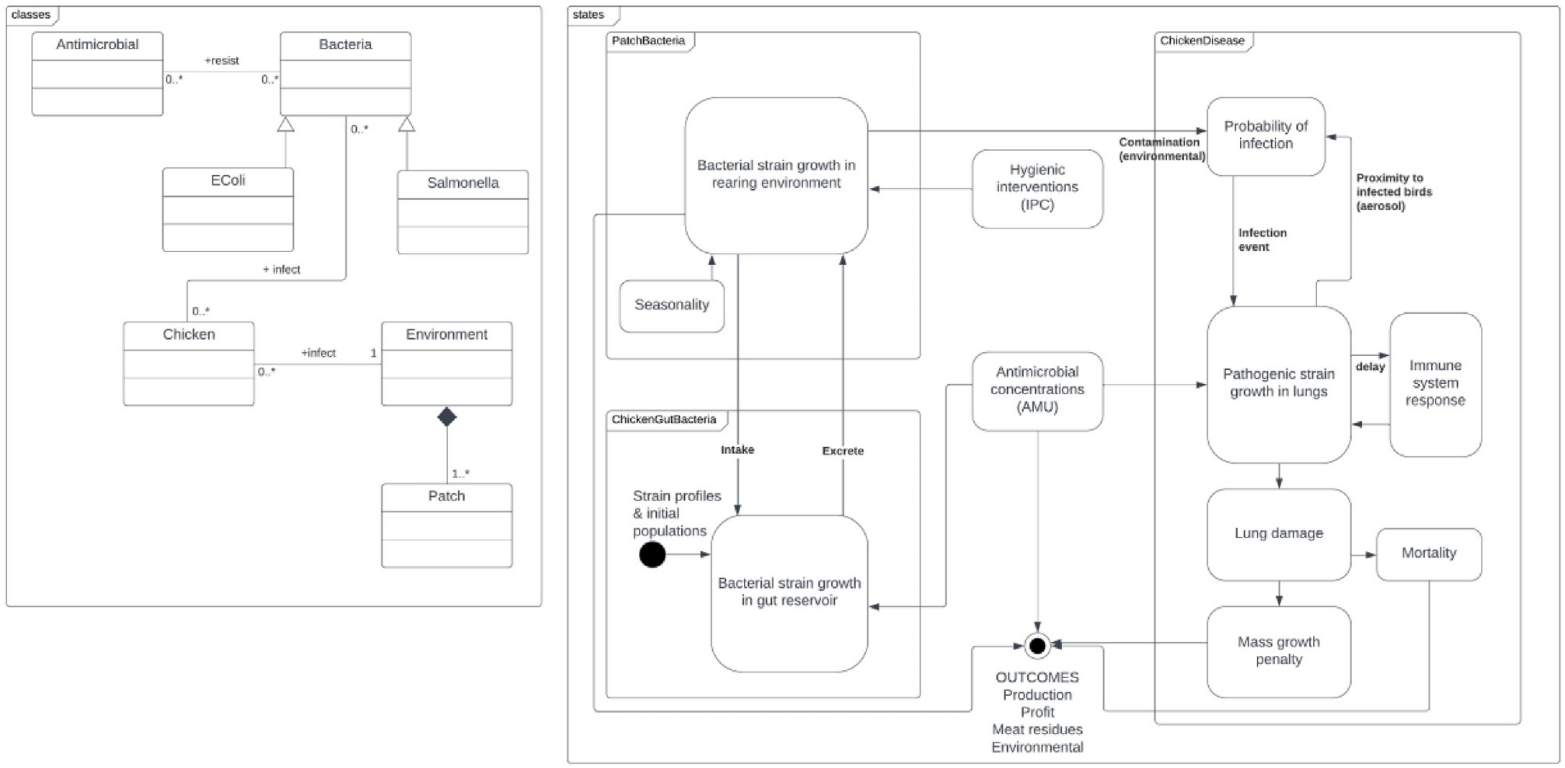
**Simplified UML class and state diagrams showing key model elements**.

Initial development aimed to elaborate a stable simulation, grounded in existing evidence, sufficient to be taken forward in participatory processes and to support initial observations about the added value of ABM in this application. Fig. 2 lays out simplified representations of model elements, with more detail available in the ODD documentation mentioned above.

Model design drew on existing mathematical modelling of AMR [28], in particular settling on Græsbøll et al.’s approach to modelling AMR in pig farming [29], which describes AMR dynamics as a function of competition between strains with varying susceptibility and fitness costs. Although this is necessarily a simplification of understandings of AMR dynamics, for example excluding horizontal transmission mechanisms [30], a competition model capable of supporting multiple bacterial strains and multiple antimicrobial agents with minimal modification offered considerable advantages in terms of representing real-world production environments in a flexible and extensible way. The clearest advantage was being able to move beyond exclusive focus on a single “bug-drug” combination, which is a clear limitation in many mathematical models in the field, with no limit to the number of bacterial strains or antimicrobial agents which our model could incorporate in principle. The competitive model also enabled relative simplicity in key modelling dynamics: bacterial strains defined exclusively in terms of growth curves according to antimicrobial concentrations; and a core equilibrium relationship ultimately governing the evolution of strains relative to each other.

Mathematical models were further deployed to add disease mechanics to the model, emphasising usefulness of representing individual agent immune responses to pathogenic bacteria and maximal simplicity. These considerations favoured mobilisation of a simplified version of Mochan et al.’s intra-host model for pneumonia in mice [31], with the model representing the complicated pathogenesis and epidemiology of colibacillosis and salmonellosis [32,33] in radically simplified terms.

The model was configured to produce a text-based output from each simulation run which summarised some of the outcomes relevant to stakeholders in economic, human health and environmental practices. An example output text is provided in Appendix A.

The team referred to practitioner experience within the author group, plus field data to improve representation of current realities in the chosen region of Senegal. For example, the “enclosure cleaning” originally included as a basic infection prevention and control (IPC) intervention which the model observer may deploy was replaced with an “add litter” intervention in response to the comment that producers rarely or never undertake cleaning during a production cycle. In addition the team analysed fresh AMUSE survey data from Senegal for model calibration purposes [34,35].

Model development culminated in testing and calibration, generating an initial parameter space (Appendix B) and the design of a set of experimental intervention strategies for evaluation over repeated simulation runs (Appendix C).

## 3 Results

Our work shows that it is possible to mobilise computer-based ABM simulation of food production systems in LMIC in ways that illustrate the relevance of AMR to the practical challenges and priorities of producers and veterinarians.

The first result is production of a stable and robust model with a high degree of flexibility in terms of potential for integrating cross-disciplinary evidence for impact on practice and policy. Local sensitivity analysis using a one-at-a-time approach [36] with Netlogo’s embedded BehaviorSpace tool suggested a viable initial parameter space, as indicated in Appendix B, capable of supporting demonstrations for stakeholders illustrating the relevance of AMR factors to their day-to-day priorities including incidence of mortality and morbidity.

**Fig. 3:**
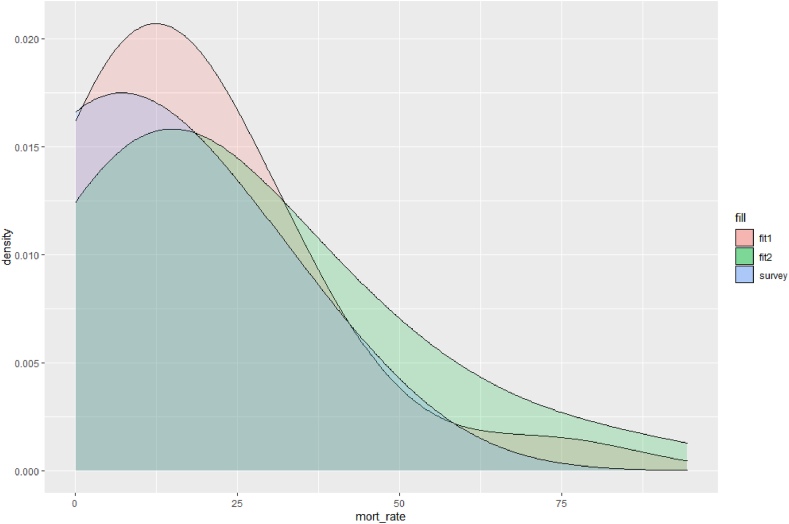
**Example density plots from exploratory calibration of model to base case (no AMU).***(Survey = estimated distribution of mortality rate from field data* [34,35]. *Fit 1 & fit 2 = distribution of mortality rates using values shown in*Appendix B*(only varying seasonal impacts on bacterial growth rates in the model environment)).*

The second result is calibration of the model environment to best-available field data. The initial parameter space was explored to suggest a set of values obtaining good-enough fit for baseline purposes. Although non-use of antimicrobial treatments in the study context is reportedly rare, with small sample sizes generating wide margins of error in estimates of applicable mortality rates, this represents the soundest empirical basis for calibrating the model. We used the BehaviorSearch tool to fit for target mean mortality with no interventions, followed by further BehaviorSpace testing to settle on a reasonable basis. Fig. 3 gives an example visualisation output from this process.

The third result is a set of *in silico* experimental observations obtained from simulation runs over the set of AMU strategies identified as being most relevant to the model setting (see Appendix C). See Table 1. As expected, this shows the advantage to broiler producers in terms of mortality reduction of using antimicrobial treatments (strategies 2–4) over purely hygiene-based (strategies 5 & 6) or no-intervention approaches (strategy 1). The prophylactic approach (strategy 4) was much less effective at reducing mortality than expected, probably because of the low antimicrobial concentrations used for experimental purposes. Meanwhile the “quick stop” strategy (strategy 2) was highly effective at reducing mortality, but at considerable externality cost in terms of non-respect of withdrawal periods prior to slaughter and associated risks to human health through meat residues [37].

**Table 1:** **Summary mortality statistics and antimicrobial residue in meat due tonon-respectof withdrawal periods, over 200 simulation runs over different AMU strategies.**

Strategy (see Appendix C)	Mortality rate (%) - mean	Mortality rate (%) - standard deviation	Estimated meat residue
No_Intervention	26.1	24.0	None
Responsive_Quick_Stop	0.2	0.3	Very high
Responsive_Complete	8.4	10.4	None
Prophylactic	15.8	17.6	None
Hygiene_responsive	27.6	25.0	None
Regular_hygiene	21.0	21.3	None
Response_quick_plus_hygiene	0.2	0.3	Very high
Response_complete_plus_hygiene	7.7	10.4	None

**Fig. 4:**
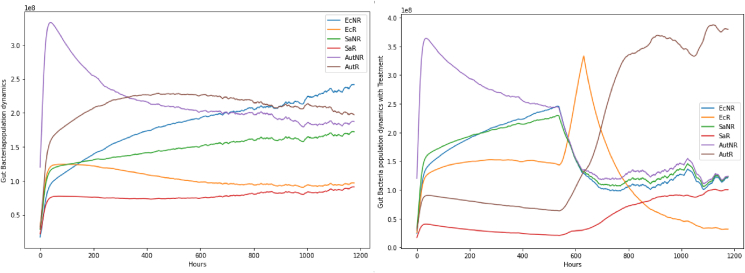
**Model populations of bacterial strains in an individual chicken over the course of simulation runs.***(In the absence of antimicrobial treatment (left), susceptible and resistant strains co-exist in equilibrium in environmental context. But periods of antimicrobial treatment are disruptive (right – treatment over c.550-650 h), conferring competitive advantage on relatively resistant strains and so tending to promote AMR generally).*

**Fig. 5:**
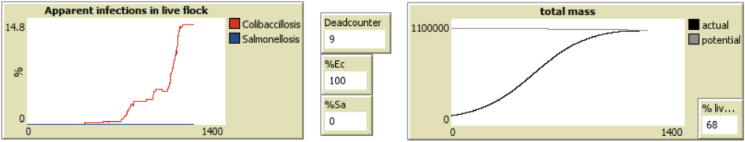
**Key model variables from a producer’s perspective.***(Apparent infections (left) may lead to the death of chickens (counted at centre), which impacts the total mass of birds (right) and therefore ultimately the value of the meat that will be sold).*

The fourth result is a set of initial observations about AMR policy in the broiler production sector in Senegal based on experience of model development and behaviour. The most significant observation is the value of addressing human behaviour, especially in market systems context given animal health challenges. Our investigation originally sought to engage with underlying resistance dynamics in the study context, for example supplying explanations of how treatments can contribute to the general problem of growing resistance (Fig. 4). This proved significantly less interesting to domain specialists than flock health surveillance for the purposes of maximising productive efficiency (Fig. 5). The utility of modelling to support policy reflections in this context therefore depends on demonstrating the relevance of AMR and AMR-related initiatives to production goals, rather than *vice versa*. For modelling purposes, each of these dimensions remained as core emergent dynamics (ODD documentation mentioned above for more detail) and so are represented here to promote understanding. In terms of observed practical significance, productive efficiency was an apparent priority because of direct association with livelihoods.

## 4 Discussion

Model development for this study was undertaken in line with best practice in mathematical modelling of AMR [28]. Our model deploys an advanced mechanistic approach (nested agent-based), incorporating stochastic elements to represent uncertainty and variability, for example in the growth rates and resistance profiles of bacterial strains, and including some initial sensitivity analysis with internal and external validation.

Care was taken to accommodate multiple bacterial strains and multiple antimicrobial agents, moving beyond the typical limitation to single organism and/or treatment perspective and address various “drug-bug” combinations. Although the model still only speaks to bacterial disease despite producers also facing viral, fungal and environmental issues, the multi-level approach enabled the initial model to address two combinations recognised as priority issues for AMR in relation to poultry rearing in Senegal and the West Africa region. Very high AMR to tetracycline have been reported in both *Escherichia coli* and *Salmonella* spp. in Senegal [13], as well as in Mali [38] and Togo [39]. Although still considerable, rates of AMR to fluoroquinolones are reported to be lower in these studies; but this is arguably of even greater concern given their important role in human health [13]. Given the lack of published data on the resistance profiles of resistant strains, it remains to be seen whether the level of detail included at strain level might be externally validated in due course, for example using surveillance data.

To our knowledge, existing similar work on AMR in broiler production focuses on flock colonisation by relevant bacteria without including pathogenic mechanics. This limitation in scope has enabled excellent work with more detail and external validation than possible for our model to date, notably in Becker et al.’s study [40], which was also based on Græsbøll and generated insights on breeds, litter renewal and stocking densities. Our gross simplification of the relationship with infection, immunity, disease and control across multiple complex categories of poultry health conditions is difficult or impossible to justify against the modelling standards applied in such existing work. However, we believe that it is a reasonable starting point for the purposes of a model aiming to demonstrate the relevance of AMR ABM to producers and policymakers in Senegal. There is in any case a general lack of *in vivo* models of relevant disease on which to call here [41].

**Fig. 6:**
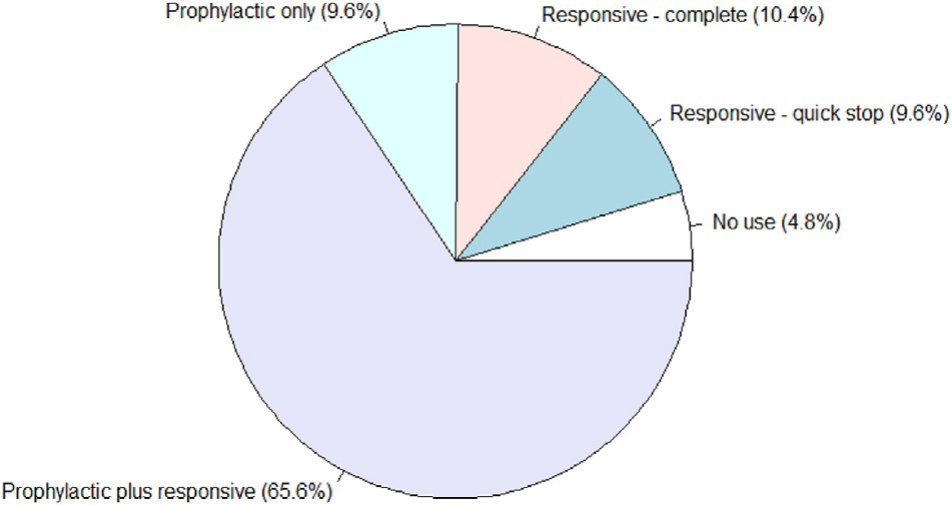
**Summary field data on prevalence in AMU stances of broiler producers in Senegal** [34,35].

The clearest example of intervention assessment from our model development to date relates to the way in which research and policy assess current AMU by poultry producers. Experimental observations from the current model are generated in the context of AMUSE field survey data on the current prevalence of AMU strategies (Fig. 6). These observations can help focus discussions on potential intervention priorities, for example deciding amongst the following three channels for policy development. Firstly, food safety regulatory action on withdrawal period compliance in meat supply chains. Secondly, producer information campaigns on the cost-ineffectiveness of prophylactic strategies. Thirdly, peer-based promotion of good practices amongst producers and/or animal health professionals.

More directly, these observations can support conceptual refinements to future rounds of data collection, for example aiming to understand specific molecules and concentrations used in prophylaxis.

Our discussion now turns from the current model status to consider prospects for future model development. Experience on modelling for decision support and policy indicates that the development process, particularly emphasising collaboration and stakeholder participation, acts a critical determinant of eventual value [42,43]. In One Health contexts like AMR, pragmatic adaptation to address partner priorities appears to be a key determinant for sustaining initiatives over time [44]. We envisage future model development to continue to emphasise participatory focus on stakeholder priorities, especially low-cost local disease surveillance for rearing programmes. This suggests some potential for wider model contributions to animal health and/or food systems policy, although we confine our detailed reflections here to AMR policy in line with study objectives.

Having established proof of concept, the next step is to engage with groups in AMR initiatives in Senegal to test assumptions about what ABM adds – especially alongside other modelling approaches – to establish a foundation for further development. It is expected that our work to date can add reflections on ABM utility in this context alongside modelling work being undertaken under the *Selecting efficient farm-level antimicrobial stewardship interventions from a One Health perspective* (SEFASI) project [45]. The SEFASI project is developing a quantitative System Dynamic model (SDM) to test intervention scenarios and *ex-ante* impact assessment of the cost-effectiveness of the interventions at farm level and beyond using a One Health approach [46]. Initial causal loop diagram development indicates a high degree of complementary between SEFASI SDM work and our work, especially offering different perspectives on the issues of farm productivity, profit and investment in good management practices [47].

One apparent strength of ABM in this context is its capacity to simulate the perspective of the people whose behaviour is understood to determine AMR-related outcomes. Most modelling involves more deterrent levels of abstraction, for example demanding system-wide overviews or concentrating on causal relationships that are assumed to be important. The observer interface of our ABM, by contrast, is designed to simulate a broiler flock rearing cycle in a way that is recognisable to producers and can help other groups develop a more detailed appreciation of their perspective. The simulation framework is flexible enough to accommodate the very wide range of dimensions relevant to assessing AMR-related interventions. Questions about how relevant dimensions have been or might be incorporated can at least help stimulate discussions about how interventions can be assessed and quantified [48]. Ideally, there is significant potential for further collaborative development of the model using participatory approaches, and perhaps even moving towards more elaborate interactive simulation or serious gaming [49].

More generally, there is an opportunity to build on mapping techniques for representing these system-level factors in terms of their composite actions and actors. This may provide a foundation to support AMR policy practice and programming through coherent integration of wider factors into future iterations of our ABM. In particular, conceptual frameworks for engaging with actor behaviour consistently across social systems is likely to prove helpful as a next step for assessing modelling priorities oriented towards evaluable intervention design [50,51]. It seems unrealistic to expect that information system improvements, for example the availability of AMR and AMU surveillance data in Senegal, will transform quickly or profoundly enough to overcome the severe limitations of ABM in terms of external validity. Similarly, there is a risk that modelling for modelling’s sake will exacerbate the existing issues of model complexity and resource-intensiveness. Instead it would be useful to use existing data and evidence, in participatory policy conversations, to focus modelling, interventions and evaluation on viable behaviour changes [52].

Our results are presented with acknowledgement of limitations. This paper presents results from proof-of-concept model development research. Its contribution is therefore conceptual, with no novel empirical or analytical insights claimed. We show the relevance and utility of ABM in this context as a reference point for future work rather than making a substantive contribution to ongoing debates at this stage.

## 5 Conclusions

This paper has presented findings from the proof-of-concept stage of research into ABM for AMR, using Senegal poultry production as a case study and aiming to inform collaborative policy development. We present some results from initial model development, with discussion focused on the potential future elaboration of this approach.

Conventional application of the terms “irrational use”, “inappropriate use” or “misuse” of antimicrobial treatments tends to underestimate the extent to which day to day AMU in LMIC agriculture addresses immediate economic goals. Senegal is well-placed, in collaboration with international partners, to contribute conceptual frameworks which can help drive closer engagement with the issues involved – including through further development and validation of future versions of the model proposed here. Many poultry producers in Senegal feel that they have little choice but to make extensive use of antibiotics, faced with the perceived alternative of disease and death driving them out of business. ABM may prove useful in this context by suggesting ways to think about the problem differently, for example the possibility that farmers may be wasting their time and money on strategies that don’t work well or the likelihood that some farming practices may be making it more difficult to fight human diseases. In terms of national policy, our model might contribute to novel thinking which considers the problem as a potential stimulus for investment in improved farming practices rather than as a competing priority.

Although we present associated results and discuss related ideas in this paper, its main contribution is to introduce the model and make the model available to wider research and policy communities. It is hoped that this will promote debate and collaboration around AMR in Senegal, as well as ABM for AMR generally.

## Author contributions

**Paul Python Ndekou, Archie Drake:** Conceptualization, Software, Original draft preparation and editing. **Paul Python Ndekou**, **Archie Drake**, **Jake Lomax**: Methodology. **Paul Python Ndekou**, **Mohamed Daly Njiemessa Nsangou**, **Archie Drake**: Validation. **Michel Dione**, **Ardiouma Faye**, **Archie Drake**, **Paul Python Ndekou**: Data curation & analysis. **Michel Dione, Jake Lomax**, **Lilian Korir**, **Elizabeth Sklar:** Writing- Reviewing and Editing. **Archie Drake**: Project Administration. **Elizabeth Sklar**, **Jake Lomax**, **Michel Dione**: Supervision. All authors contributed to the article and approved the submitted version.

## Funding source

Michel Dione and Ardiouma Faye were supported by Swedish International Development Cooperation Agency (SIDA) grant number APH002001.

## Availability of data and materials

Data used for this paper is available from original sources as detailed in citations (see [35]). Model code and ODD documentation have been published in a public repository as cited in the text (https://github.com/archiedrake/senegalabm.git).

## Ethics approval and consent to participate

Not applicable.

## Consent for publication

Not applicable.

## Conflict of interest statement

The authors whose names are listed immediately below certify that they have NO affiliations with or involvement in any organization or entity with any financial interest (such as honoraria; educational grants; participation in speakers’ bureaus; membership, employment, consultancies, stock ownership, or other equity interest; and expert testimony or patent-licensing arrangements), or non-financial interest (such as personal or professional relationships, affiliations, knowledge or beliefs) in the subject matter or materials discussed in this manuscript.

Paul Python Ndekou; Jake Lomax; Lilian Korir; Elizabeth Sklar.

The authors whose names are listed immediately below report the following details of affiliation or involvement in an organization or entity with a financial or non-financial interest in the subject matter or materials discussed in this manuscript.

Archie Drake reports an interest in the UK Fleming Fund. He is contracted as an independent consultant by Itad Ltd which is contracted by the UK Department of Health and Social Care to undertake the independent evaluation of the Fleming Fund. This is not a material conflict since the Fleming Fund is only mentioned in passing as a notable AMR initiative in Senegal which is not controversial.

Michel Dione and Ardiouma Faye report an interest in the SEFASI project. They are, respectively, employed by and affiliated with the International Livestock Research Institute (ILRI) which is funded by SIDA to implement the SEFASI project (see Funding Statement). This is not a material conflict because no view of SEFASI is presented beyond descriptions of the work as supported by published material and the opinion that the model presented in this paper will be useful to advance SEFASI objectives.

## Figures and Tables

**Table:** 

Variable	Description	Range (min – max)	Sensitivity	Calibrated value (fit to field data)
patchgrowthprop	Proportion determining bacterial growth rate in litter relative to intestines	0.001 – 0.002	High	0.0012
season	Term denoting temperature/humidity affecting bacterial growth in litter	−50 – 5	High	−20/0
infectprobs	Term controlling how likely infections are to occur	0.001 – 0.01	High	0.01
infectscale	Term controlling scale of infections	100,000–10,000,000	Medium	6,000,000
lunggrowthprop	Proportion determining bacterial growth in lung relative to intestines	0.08 – 0.5	Medium	0.1
lungmechclearrate	Rate at which the innate immune system initially controls the bacteria in the lung through mechanical clearance via cilia and mucus	1000 – 10,000	Low	1000
immuneinfluxrate	Rate at which phagocyte immune cells initially influx to lungs	10,000 – 100,000	Low	10,000
lungphagoclearrate	Rate at which the innate immune system initially controls the bacteria in the lung through phagocytosis by alveolar macrophages	1,000,000 – 10,000,000	Low	1,000,000
phagoinhib	Term for inhibition of phagocyte phagocytosis	1.0E-13 – 1.0E-10	Low	1.0E-13
damageincreaserate	Rate at which damage to the epithelium of the lungs increases proportional to pathogenic strain populations in the lungs	1.0E-11 – 1.0E-8	High	1.0E-11
damagerepairrate	Rate at which damage to the epithelium of the lungs decreases through body repair	– 0.1	Medium	0.1
moveprob	Probability that chicken changes location in a time step (1 h)	– 0.4	Medium	0.3

## Appendix A example simulation run output report

Strategy: Prophylactic.

**ECONOMIC OUTCOMES.**

**1. Production.**

588.6 kg meat produced.

58.86 % efficiency vs theoretical maximum.

**2. Income.**

706,318 FCFA total income.

(Per kilo price 1200 FCFA)

**3. Expenses.**

943,363 FCFA total expenses.

(Chick purchase plus feed over time plus medications plus labour)

500,000 on chicks at FCFA 1000 each.

429,159 on feed calculated as per gram of mass produced including birds that died and excluding start weight - FCR 1.6 and FCFA 300 per kilo (FCFA 15,000 per 50 kg)

Medications costs included (assuming FCFA 0.01 to achieve 1 unit of concentration per bird in each case):

4203 on Tetracycline.

0 on fluoroquinolones.

10,000 on labour - fixed 10,000 plus 5,000 per adding of litter (0 - times)

**4. Profit.**

−237,044 FCFA gross profit.

**HUMAN HEALTH - RELATED OUTCOMES.**

**1. AB residue in meat.**

0 units of antimicrobial medicine concentration in meat due to non-respect of withdrawal period of 10 days.

**2. Resistant pathogenic bacteria passed to meat supply chain.**

1,329,899,965,716 grand total.

Including 1,255,523,120,145 resistant E Coli strain and 74,376,845,571 resistant Salmonella strain.

Including 158,588,728,475 in intestines and 1,171,311,237,240 in lungs.

**3. Exposure of producers & wider community to resistant pathogenic bacteria.**

5,854,195,246,318,694 grand total patch EcR & SaR over whole production cycle.

**ENVIRONMENT - RELATED OUTCOMES.**

**1. Waste disposal from cage cleaning - resistant pathogenic bacteria.**

22,912,826,361,185 resistant pathogenic bacteria emitted to environment from final cleaning.

**2. Carcass disposal - resistant pathogenic bacteria.**

1,765,553,227,550 resistant pathogenic bacteria emitted to environment from carcass disposal.

## Appendix C AMU/IPC intervention strategies for ABM of Senegal broiler production

Producer decision-making about using medicines (primary - tetracycline, secondary - fluoroquinolones) or hygienic improvements (enclosure cleaning) given observations over time.

**1. No intervention.**

Producer takes no action.

**2. Responsive quick stop.**

Producer uses medicines in response to apparent infections and withdraws them immediately if apparently effective.

If 3 or more birds apparently diseased (lung damage >20 %), immediately start medium dose of primary antibiotic (concentration 10). If still 3 or more birds apparently diseased after 48h, escalate primary to high dose (concentration 40). If still 3 or more birds apparently diseased after further 48h, add high dose of secondary antibiotic (concentration 40).

Treatment only stops – immediately – if no birds apparently diseased.

**3. Responsive complete.**

Producer uses medicines in response to apparent infections and continues with full course regardless of effect.

If 3 or more birds apparently diseased (lung damage >20 %), immediately start medium dose of primary antibiotic (concentration 10) over 7 days (168h). If still 3 or more birds apparently diseased after this period, switch to high dose of secondary antibiotic (concentration 40) over 3 days (72h).

Treatment continues for these periods regardless of apparent disease; but withdrawal period of 7 days respected prior to slaughter.

**4. Prophylactic.**

Producer uses medicines to try to prevent infections, and/or for growth promotion, stress reduction purposes.

Low dose of primary antibiotic (concentration 1) from the start.

Withdrawal period of 7 days respected prior to slaughter.

**5. Hygiene responsive.**

Producer uses hygiene in response to apparent infections. Check enclosure every 3 days, if 3 or more birds apparently diseased (lung damage >20 %) then add litter.

**6. Regular hygiene.**

Producer follows hygiene routine. Add litter every 7 days.

**7. Responsive quick stop** **+** **hygiene.**

Combination of interventions 2 and 5.

**8. Responsive complete** **+** **reg. hygiene.**

Combination of interventions 3 and 6.

## References

[1] Murray C.J., Ikuta K.S., Sharara F., Swetschinski L., Aguilar G.R., Gray A. (2022). Global burden of bacterial antimicrobial resistance in 2019: a systematic analysis. Lancet.

[2] World Health Organization (2015). https://www.who.int/publications-detail-redirect/9789241509763.

[3] World Health Organization (2017). https://www.who.int/news-room/questions-and-answers/item/one-health.

[4] Hedman H.D., Vasco K.A., Zhang L. (2020). A review of antimicrobial resistance in poultry farming within low-resource settings. Animals (Basel).

[5] Food and Agriculture Organization (2021 https://www.fao.org/documents/card/en?details=cb5545en).

[6] Caudell M.A., Dorado-Garcia A., Eckford S., Creese C., Byarugaba D.K., Afakye K. (2020). Towards a bottom-up understanding of antimicrobial use and resistance on the farm: a knowledge, attitudes, and practices survey across livestock systems in five African countries. PLoS One.

[7] Caudell M.A., Kiambi S., Afakye K., Koka E., Kabali E., Kimani T. (2022). Social-technical interventions to reduce antimicrobial resistance in agriculture: evidence from poultry Farmer Field Schools in Ghana and Kenya. JAC-Antimicrobial Resistance.

[8] Food and Agriculture Organization of the United Nations (2021). https://www.fao.org/senegal/actualites/detail-events/zh/c/1392917/.

[9] Dione M.M., Ieven M., Garin B., Marcotty T., Geerts S. (2009). Prevalence and antimicrobial resistance of Salmonella isolated from broiler farms, chicken carcasses, and street-vended restaurants in Casamance, Senegal. J. Food Protect..

[10] Fall-Niang N.K., Sambe-Ba B., Seck A., Deme S.N., Wane A.A., Bercion R. (2019). Antimicrobial resistance profile of Salmonella isolates in chicken carcasses in dakar, Senegal. Foodborne Pathogens and Disease.

[11] Vounba P. (2019).

[12] Vounba P., Kane Y., Ndiaye C., Arsenault J., Fairbrother J.M., Bada Alambédji R. (2018). Molecular characterization of Escherichia coli isolated from chickens with colibacillosis in Senegal. Foodborne Pathogens and Disease.

[13] Vounba P., Arsenault J., Bada-Alambédji R., Fairbrother J.M. (2019). Prevalence of antimicrobial resistance and potential pathogenicity, and possible spread of third generation cephalosporin resistance, in Escherichia coli isolated from healthy chicken farms in the region of Dakar, Senegal. PLoS One.

[14] African Society for Laboratory Medicine (2022). https://aslm.org/wp-content/uploads/2022/09/ASLM_MAAP-Policy-Brief_Embargoed-until-15-Sept-6AM-GMT.pdf?x26552.

[15] African Society for Laboratory Medicine (2022). https://aslm.org/wp-content/uploads/2023/07/AMR_REPORT_SENEGAL.pdf?x89467.

[16] Lhermie G., Ndiaye Y., Rushton J., Raboisson D. (2022). Economic evaluation of antimicrobial use practices in animal agriculture: a case of poultry farming. JAC-Antimicrobial Resistance.

[17] Painter C., Faradiba D., Chavarina K.K., Sari E.N., Teerawattananon Y., Aluzaite K. (2023). A systematic literature review of economic evaluation studies of interventions impacting antimicrobial resistance. Antimicrob. Resist. Infect. Control.

[18] Food and Agriculture Organization of the United Nations Reduce the Need for Antimicrobials on Farms 2023. https://www.fao.org/antimicrobial-resistance/news-and-events/news/news-details/en/c/1629402/.

[19] Ly C. Aviculture et Covid-19 au Sénégal Situation et perspectives Note d’information et d’analyse, 2020. https://www.ipar.sn/IMG/pdf/covid19_aviculture_au_senegal_ipar_nov_2020.pdf.

[20] Ba K., Diouf A.D., Ba M., Ly C. (2022). Les succès de l’aviculture commerciale en Afrique sub-saharienne : Le cas du Sénégal. IPAR - Sénégal.

[21] Hammond RA. Considerations and Best Practices in Agent-Based Modeling to Inform Policy. In: Committee on the Assessment of Agent-Based Models to Inform Tobacco Product Regulation; Board on Population Health and Public Health Practice; Institute of Medicine; Wallace R, Geller A, Ogawa VA, editors. Assessing the Use of Agent-Based Models for Tobacco Regulation. Washington (DC): National Academies Press (US); 2015 Jul 17. Appendix A. Available from: https://www.ncbi.nlm.nih.gov/books/NBK305917/26247084

[22] Steinbacher M., Raddant M., Karimi F., Camacho Cuena E., Alfarano S., Iori G. (2021). Advances in the agent-based modeling of economic and social behavior. SN Bus Econ.

[23] Sklar E. (2007). NetLogo, a multi-agent simulation environment. Artif. Life.

[24] Mshana S.E., Sindato C., Matee M.I., Mboera L.E.G. (2021). Antimicrobial use and resistance in agriculture and food production systems in Africa: a systematic review. Antibiotics.

[25] Ducrot C., Hobeika A., Lienhardt C., Wieland B., Dehays C., Delabouglise A. (2021). Antimicrobial resistance in africa—how to relieve the burden on family farmers. Emerg. Infect. Dis..

[26] Grimm V., Berger U., Bastiansen F., Eliassen S., Ginot V., Giske J. (2006). A standard protocol for describing individual-based and agent-based models. Ecol. Model..

[27] Grimm V., Railsback S.F., Vincenot C.E., Berger U., Gallagher C., DeAngelis D.L. (2020). The ODD protocol for describing agent-based and other simulation models: a second update to improve clarity, replication, and structural realism. J. Artif. Soc. Soc. Simul..

[28] Birkegård A.C., Halasa T., Toft N., Folkesson A., Græsbøll K. (2018). Send more data: a systematic review of mathematical models of antimicrobial resistance. Antimicrob. Resist. Infect. Control.

[29] Græsbøll K., Nielsen S.S., Toft N., Christiansen L.E. (2014). How fitness reduced, antimicrobial resistant bacteria survive and spread: a multiple pig - multiple bacterial strain model. PLoS One.

[30] Baker M., Hobman J.L., Dodd C.E.R., Ramsden S.J., Stekel D.J. (2016). Mathematical modelling of antimicrobial resistance in agricultural waste highlights importance of gene transfer rate. FEMS (Fed. Eur. Microbiol. Soc.) Microbiol. Ecol..

[31] Mochan E., Swigon D., Ermentrout G.B., Lukens S., Clermont G. (2014). A mathematical model of intrahost pneumococcal pneumonia infection dynamics in murine strains. J. Theor. Biol..

[32] Kabir S.M.L. (2010). Avian colibacillosis and salmonellosis: a closer look at epidemiology, pathogenesis, diagnosis, control and public health concerns. Int. J. Environ. Res. Publ. Health.

[33] Kathayat D., Lokesh D., Ranjit S., Rajashekara G. (2021). Avian pathogenic Escherichia coli (APEC): an overview of virulence and pathogenesis factors, zoonotic potential, and control strategies. Pathogens.

[34] Faye A. Connaissances, attitudes et pratiques des éleveurs de volailles sur l’usage des antibiotiques en zone péri urbaine de Dakar, 2022. https://cgspace.cgiar.org/handle/10568/126952.

[35] Emes E., Faye A., Naylor N., Belay D., Ngom B., Fall A.G. (2023).

[36] Ligmann-Zielinska A., Siebers P.-O., Magliocca N., Parker D.C., Grimm V., Du J. (2020). mixed-method pathways for sensitivity analysis of agent-based models. JASSS.

[37] Mensah S.E.P., Koudandé O.D., Sanders P., Laurentie M., Mensah G.A., Abiola F.A. (2014). Antimicrobial residues in foods of animal origin in Africa: public health risks. Rev Sci Tech.

[38] Sidibé S., Traoré A.B., Koné Y.S., Fané A., Coulibaly K.W., Doumbia A.B. (2019). Antibiorésistance des souches de <em>Salmonella gallinarum</em> isolées en aviculture moderne en zones périurbaines au Mali. Revue d’élevage et de médecine vétérinaire des pays tropicaux.

[39] Bedekelabou A.P., Talaki E., Dolou M., Diouf A., Alambedji R.B. (2020). Antibiotic resistance of enterobacteria (Escherichia coli, Klebsiella spp. and Salmonella spp) isolated from healthy poultry and pig farms in peri-urban area of Lome. Togo. AJMR.

[40] Becker E., Correia-Carreira G., Projahn M., Käsbohrer A. (2022). Modeling the impact of management changes on the infection dynamics of extended-spectrum beta-lactamase-producing Escherichia coli in the broiler production. Microorganisms.

[41] Kromann S., Jensen H.E. (2022). In vivo models of Escherichia coli infection in poultry. Acta Vet. Scand..

[42] Calder M., Craig C., Culley D., Cani R., Donnelly C., Douglas R. (2018). Computational Modelling for Decision-Making: where, Why, what, Who and How. R. Soc. Open Sci..

[43] Gilbert N., Ahrweiler P., Barbrook-Johnson P., Narasimhan K.P., Wilkinson H. (2018). Computational modelling of public policy: reflections on practice. JASSS.

[44] Abbas S.S., Shorten T., Rushton J. (2021). Meanings and mechanisms of One Health partnerships: insights from a critical review of literature on cross-government collaborations. Health Pol. Plann..

[45] Zannou O., Faye P.A., Dione M.M. (2022). Choisir des interventions efficaces de gestion des antimicrobiens au niveau de la ferme du point de vue de l’approche une Seule Santé : Cas d’étude du Sénégal. ILRI.

[46] ILRI (2022). https://www.ilri.org/news/new-project-seeks-improve-farm-level-antimicrobial-use-senegal-through-one-health-approach.

[47] Aboah J., Ngom B., Emes E., Fall A.G., Seydi M., Faye A. (2023). Mapping the effect of antimicrobial resistance in poultry production in Senegal: an integrated system dynamics and network analysis approach. Front. Vet. Sci..

[48] Naylor N.R., Lines J., Waage J., Wieland B., Knight G.M. (2020). Quantitatively evaluating the cross-sectoral and One Health impact of interventions: a scoping review and case study of antimicrobial resistance. One Health.

[49] Bakhanova E., Garcia J.A., Raffe W.L., Voinov A. (2020). Targeting social learning and engagement: what serious games and gamification can offer to participatory modeling. Environ. Model. Software.

[50] Lomax J. (2018).

[51] Lomax J. (2022).

[52] McKernan C., Benson T., Farrell S., Dean M. (2021). Antimicrobial use in agriculture: critical review of the factors influencing behaviour. JAC-Antimicrobial Resistance.

